# Vision–Language Models in Ultrasound Imaging: A Comprehensive Review

**DOI:** 10.3390/jimaging12100493

**Published:** 2026-10-07

**Authors:** Zeyuan Tang, Shukang Zhang, Huanjun Wang, Wei Shao, Wentao Kong, Daoqiang Zhang, Peng Wan

**Affiliations:** 1 College of Artificial Intelligence, Nanjing University of Aeronautics and Astronautics, Nanjing 211106, China; tangzeyuan@nuaa.edu.cn (Z.T.); skzhang@nuaa.edu.cn (S.Z.); huanjunwang@nuaa.edu.cn (H.W.); shaowei20022005@nuaa.edu.cn (W.S.); dqzhang@nuaa.edu.cn (D.Z.); 2 Department of Ultrasound, The Affiliated People’s Hospital of Jiangsu University, Zhenjiang 212003, China; breezewen@163.com

**Keywords:** multimodal learning, ultrasonic imaging, vision–language models

## Abstract

Ultrasound vision–language models (US-VLMs) must interpret operator-dependent images and dynamic examinations while linking visual evidence to clinical language. This review synthesizes 32 formally published studies identified through a three-database search. We apply an operational three-axis taxonomy: the interaction axis identifies the primary image–text computation, the learning axis records ultrasound-specific training objectives, and grounding granularity specifies the finest documented visual–linguistic correspondence. Study-level assignments and rationales make these distinctions reproducible. We compare task-specific results without ranking models across incompatible cohorts, endpoints, and metrics. Dataset analysis separates acquisition volume from independent clinical diversity, and a seven-domain risk-of-bias appraisal identifies limitations in reporting of patient-level partitioning and development–test overlap. We also distinguish retrospective performance, including external-cohort results, from evidence that assistance improves clinical decisions or workflows. Current systems support diverse ultrasound tasks, but clinical claims require evidence grounding, uncertainty assessment, and validation matched to the intended use.

## 1. Introduction

Ultrasound is widely used because it offers real-time, portable imaging without ionizing radiation [1]. Its interpretation remains sensitive to speckle, acoustic shadowing, limited soft-tissue contrast, and operator technique [2]. Conventional deep learning can address defined tasks such as segmentation and detection [3], but changes in anatomy, devices, and acquisition protocols may reduce performance [4].

Vision–language models (VLMs) connect visual inputs with natural-language representations through matching, fusion, or generation [5]. In medical imaging, textual supervision can support retrieval and task-specific interpretation [6]. For ultrasound, language may link ambiguous visual cues to descriptors such as echogenicity, lesion margin, and vascularity. These links remain dependent on the quality of image–text correspondence and on whether the relevant anatomy is visible.

We use the term ultrasound vision–language model (US-VLM) for a system with an explicit image–text interaction in its ultrasound task, rather than for any model that combines images with ancillary data. Section 2.2 specifies the full eligibility criteria.

Ultrasound merits separate review because dynamic acquisition, partial views, and weak frame–report correspondence affect what visual evidence a model can use. Prompt-based adaptation may reduce task-specific annotation needs [7], while free-form outputs introduce a need to check whether clinical statements are supported by the examination [8]. Compared with broad multimodal biomedical AI reviews [9], our ultrasound-specific framework separates the principal image–text interaction, ultrasound-trained objective, and finest documented grounding level. This makes frame–report mismatch and local evidence requirements explicit without treating task categories as performance rankings.

We synthesize 32 published US-VLM studies using an operational taxonomy and assess the data, evaluation evidence, and clinical limits of their reported results. Our contributions are:

- We code each study along the interaction axis, learning axis, and grounding granularity, with explicit assignment rules and study-level rationales.
- We compare model families by task and evidence type, separating dataset size from independent clinical diversity and appraising study-level risk of bias.
- We identify reliability and validation requirements that follow from the reported evidence, including visual grounding, uncertainty assessment, and prospective workflow evaluation.

## 2. Review Methodology

**Figure 1:**
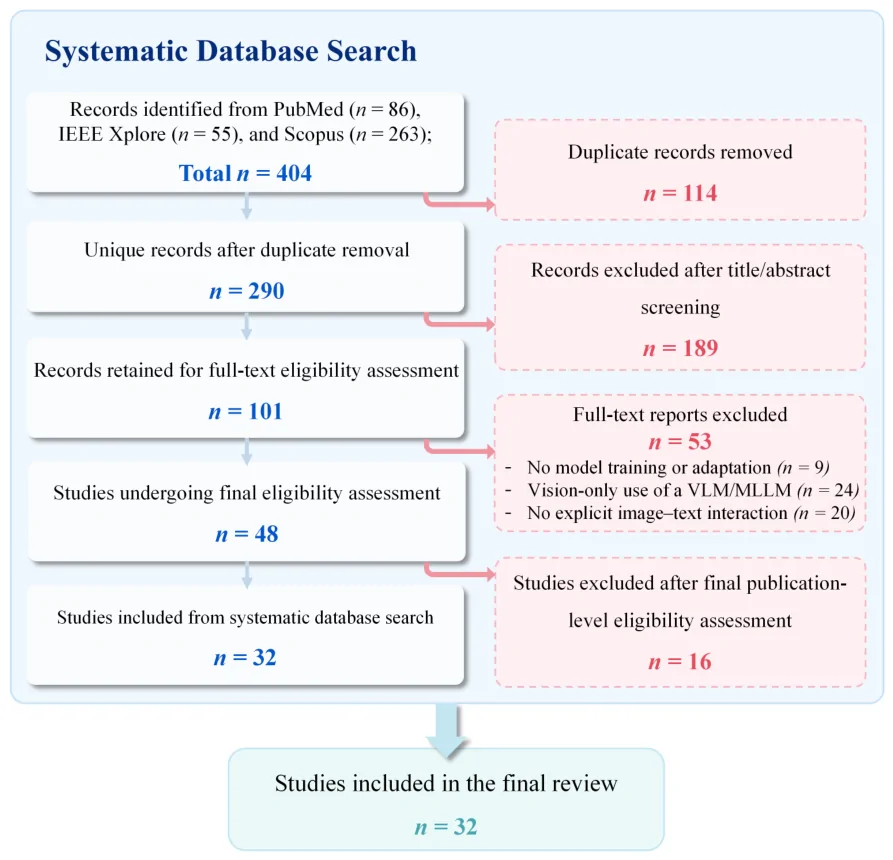
Database search, deduplication, title/abstract screening, full-text screening, and final functionality-based inclusion of the 32 studies. Blue denotes records retained at each screening stage, red denotes excluded records, and teal denotes the final included studies.

This review combines a reproducible three-database search with a functionality-based full-text screen of the ultrasound vision–language literature. The search, deduplication, screening, and inclusion stages are summarized in Figure 1.

### 2.1. Literature Search

PubMed, IEEE Xplore, and Scopus were searched on 28 September 2026 for articles published from 1 January 2021 through 30 June 2026. The latter date is the publication cutoff, not the search execution date. The ultrasound block included ultrasound, ultrasonograph*, sonograph*, echograph*, echocardiograph*, POCUS, endoscopic and intravascular ultrasound, EUS, IVUS, CEUS, and related terms. The vision–language block included vision–language, visual–language, VLM, multimodal large language model (MLLM), image–text model, CLIP in an image–text context, and named multimodal model families. The blocks were combined with AND; PubMed used Title/Abstract, IEEE Xplore used All Metadata, and Scopus used TITLE-ABS-KEY. Appendix A gives the complete database-specific strings and date restrictions (Listings A1–A3).

The executed exports contained 86 PubMed, 55 IEEE Xplore, and 263 Scopus records (404 in total; Table 1). All three databases were searched with the same publication window of 1 January 2021 through 30 June 2026; for IEEE Xplore and Scopus, the year filter was combined with record-level cutoff checking because a year filter alone cannot implement 30 June 2026.

### 2.2. Eligibility Criteria

A VLM was defined by function: ultrasound images, frames, or videos and natural-language information had to interact within an identifiable computation, such as image–text alignment, cross-attention, prompt-conditioned recognition, region–phrase grounding, or ultrasound-conditioned language generation. CLIP-type models qualified when both visual and textual representations participated in an ultrasound task. Using only the visual encoder of a pretrained VLM, or using text merely as labels, metadata, or an independent post-processing narration, did not qualify. The study had to be original research with a complete full text, dedicated to ultrasound as the sole imaging modality: the explicit visual input had to be ultrasound, and task-specific training or adaptation data had to be restricted to ultrasound, without mixing any other medical imaging modality (e.g., CT, MRI, or radiography) into training. The included studies were required to include complete task-specific training or fine-tuning of the reported model, so that zero-shot-only evaluations of unchanged general-purpose MLLMs on ultrasound images were excluded. Only formally published journal or conference articles were eligible, and preprints were excluded during screening.

### 2.3. Study Selection

Duplicate-record removal eliminated 114 of the 404 exported records, leaving 290 unique records. Title and abstract screening excluded 189 records, leaving 101 reports for full-text assessment. Of these, 53 were excluded because they did not meet the predefined eligibility criteria. The remaining 48 candidate studies then underwent a final publication-level eligibility assessment, after which 16 were excluded. The resulting 32 studies constituted the final evidence corpus for this review.

### 2.4. Methodological Quality and Risk-of-Bias Assessment

We performed a structured, report-based appraisal of the principal evaluated endpoint in each of the 32 studies, including a headline assisted-reader claim when one was reported. Seven domains were assessed: D1, case selection and eligibility; D2, reference-standard or annotation provenance; D3, patient- or examination-level partitioning; D4, development–test overlap and leakage safeguards; D5, independence of the test set from model, prompt, and threshold selection; D6, appropriateness of the evaluation unit, metrics, and uncertainty analysis; and D7, completeness of reporting for the principal endpoint. Each domain was rated L (low risk), H (high risk), or U (unclear); NA was reserved for a genuinely inapplicable domain. L required a directly reported safeguard or appropriate analysis; H required a reported design feature capable of biasing the stated estimate; and missing information was rated U rather than presumed to represent either a safeguard or a confirmed flaw. Overall risk was H if any applicable domain was H, otherwise U if any domain was U, and L only if every applicable domain was L. No numerical quality score was calculated. D5 concerns test-set use during development; external-site breadth and clinical diversity were assessed separately in Section 5. Table A1 and Table A2 report the study-level profiles and the basis for each overall judgment.

The appraisal yielded two L, two H, and 28 U overall judgments. The H judgments concern the fixed order of unaided and assisted reading of the same cases in the mid-trimester examination study and generated answers used as benchmark references in LLAUS [10,11]; they do not describe every endpoint in those papers. Patient- or examination-level partitioning was unclear in 18 studies, and development–test overlap safeguards were unclear in 21. These U judgments reflect limits of the reported methods, not demonstrated leakage. The two L judgments are confined to the reported FTC-Net diagnostic cohorts and XBusNet BLU segmentation evaluation; they do not establish general clinical utility [12,13]. Ratings were not used to rank task performance across studies.

### 2.5. Data Extraction

For each included article, we recorded publication metadata, imaging modality, image–text mechanism, task, dataset or cohort, and study-specific outcomes. We separated unique clinical units (patients, participants, examinations, cases, or records) from nested images, frames, videos, reports, masks, boxes, instructions, and image–text pairs. We also recorded reported institutions, external test sources, geography, vendors, operators, protocols, population characteristics, and center-wise allocation to development and testing. A pooled multicenter cohort, public dataset, or large acquisition count was not treated as independent external validation. Counts retain the unit reported by each study; related sources were not summed as independent patients. Model parameter counts were not pooled because the reported totals cover different components.

For temporal assessment, we recorded the acquisition unit, actual model input, treatment of frame order, temporal operator or objective, and any sequence-specific evaluation. Explicit temporal modeling required ordered frames and evidence that temporal order was preserved or tested through an identifiable component, objective, or evaluation. Video provenance, random frame sampling, and unordered multi-view aggregation alone did not meet this rule. Technical limitations in the synthesis describe the reported evidence boundary rather than an inferred outcome.

### 2.6. Operational Taxonomy Coding

The coding unit was the ultrasound-trained system proposed by each of the 32 included papers, rather than every baseline, downstream test, or external model used to prepare training data. We examined each included study for the image–text computation that produces its principal ultrasound endpoint, the ultrasound-specific training objective, and the finest image–text correspondence explicitly trained or evaluated. Table 2 gives the decision rules; Table A1 and Table A2 report every assignment with a short mechanism-based rationale. These assignments are descriptive literature codes, not claims that the cited systems are clinically validated.

The interaction axis has one primary interaction code: I1, separate-encoder matching by visual and language embeddings; I2, fusion-based prediction from joint image–text features to a non-linguistic class, measurement, box, or mask; or I3, ultrasound-conditioned language generation. We selected the code from the computation producing the principal reported endpoint, rather than the name of a pretrained backbone or a downstream use case. A frozen language decoder still counts as I3 when a trained ultrasound-specific connector conditions its text output. For multistage systems, the assignment tables name a secondary mechanism where it could otherwise cause ambiguity.

The learning axis permits multiple learning codes for ultrasound-specific training: L1 denotes image–text alignment or matching, L2 supervised non-linguistic task prediction, and L3 conditional text generation or visual instruction tuning. A pretrained backbone’s original objective is not assigned as a new ultrasound-specific learning code. Few-shot sampling, prompt learning, and parameter-efficient fine-tuning are adaptation strategies recorded in the rationale rather than mutually exclusive learning classes. Grounding granularity (G0–G3) records the finest explicit visual–linguistic correspondence: global image/video/examination to text (G0), frame/view to sentence or descriptor (G1), lesion/region/box to phrase or description (G2), and text-conditioned pixel/mask prediction (G3). The grounding level requires a documented training or evaluation path; attention visualizations, a segmentation module independent of text, and a mask obtained from a frozen post-processor do not alone establish G2 or G3.

## 3. Ultrasound-Specific Challenges and Taxonomy of US-VLMs

### 3.1. Ultrasound-Specific Acquisition and Supervision Challenges

Ultrasound acquisition and supervision differ from those of static cross-sectional imaging. Five characteristics shape US-VLM design: speckle, acoustic artifacts, and limited soft-tissue contrast [25,26,27]; operator-dependent views and partially visible anatomy [28]; dynamic inputs spanning frames, cine loops, Doppler sequences, sweeps, and examinations; weak correspondence between individual frames and examination-level reports; and the close coupling of image acquisition with interpretation. These characteristics determine which visual evidence a model can access and how reliably language supervision identifies that evidence.

### 3.2. Three-Axis Taxonomy: Interaction Axis, Learning Axis, and Grounding Granularity

The three axes answer different questions: how ultrasound and language interact (I1–I3), which ultrasound-specific objectives train the interaction (L1–L3), and how locally visual evidence is tied to language (G0–G3). The codes and their observable criteria are defined in Section 2.6 and Table 2. A study can carry several learning codes but only one primary interaction code. The 32 study-level assignments and their methodological rationales appear in Table A1 and Table A2.

### 3.3. Relationships Among the Three Axes

The axes are related without being interchangeable. Contrastive dual encoders can support study-level retrieval and may also align individual views or frames to sentences, as in Sonomate and FCUFormer [15,23]. Conversely, a language-model backbone does not make a classification-head system a language generator: EchoReason and MMVL predict disease categories from fused features [16,17]. Spatial claims require an additional distinction. Grounding DINO-US-SAM learns text-guided box localization, while its downstream SAM2 mask decoder remains frozen; VLS predicts lesion boxes together with reports before SAM produces masks [18,29]. Both are coded G2 rather than attributing independent pixel-level image–text training to the frozen mask stage.

### 3.4. Acquisition Challenges and Clinical Reliability Pathways

**Figure 2:**
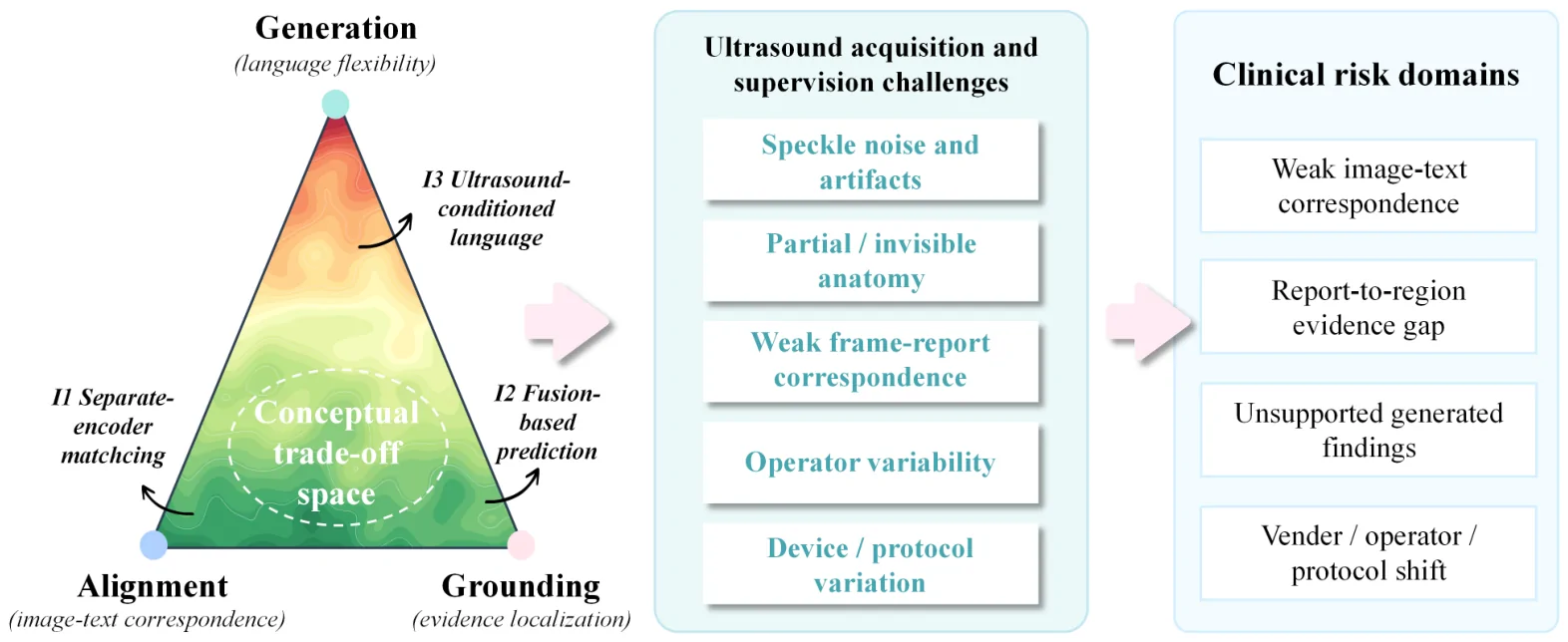
Qualitative clinical reliability framework for US-VLMs. The figure links conceptual model capability dimensions, ultrasound acquisition and supervision challenges, and four clinical reliability pathways: weak image–text correspondence, report-to-region evidence gaps, unsupported generated findings, and vendor/operator/protocol distribution shift. These pathways are qualitative and non-additive; the figure does not define a clinical-risk score or imply a cross-study safety ranking.

The acquisition and supervision challenges comprise speckle noise and artifacts, partial or invisible anatomy, weak frame–report correspondence, operator variability, and device or protocol variation. They can contribute to four qualitative clinical reliability risk pathways: weak image–text correspondence, a report-to-region evidence gap, unsupported generated findings, and vendor/operator/protocol distribution shift. These pathways are not additive or independently measured components of a clinical-risk score. Image–text matching can assess correspondence; phrase-to-region localization can assess grounding; expert-reviewed statement factuality can assess generation; and cross-site performance or calibration can assess distribution shift. The included studies do not apply a common protocol for these endpoints, so a cross-study risk total or safety ranking would be unwarranted. Figure 2 sketches the conceptual links; Section 6 separates direct observations from proxy and unquantified evidence.

### 3.5. Learning Behavior and Optimization Trade-Offs in US-VLMs

The operational learning codes distinguish ultrasound-specific image–text alignment (L1), supervised prediction (L2), and conditioned language generation (L3); spatial grounding is coded separately as G2 or G3 when the source reports an explicit local image–text path. In the 32-paper corpus, EchoCLIP, FetalCLIP, SonoEye and EchoPrime align ultrasound images or videos with reports or other natural language [14,19,30,31]. An illustrative CLIP-style, bidirectional batch objective is

(1) $$\begin{array}{cc} L_{v\to t} & =-\frac{1}{N}\sum_{i=1}^{N}\log\frac{\exp(s\left(v_{i},t_{i}\right)/\tau )}{\sum_{j=1}^{N}\exp\left(s\left(v_{i},t_{j}\right)/\tau \right)},\\ L_{t\to v} & =-\frac{1}{N}\sum_{i=1}^{N}\log\frac{\exp(s\left(t_{i},v_{i}\right)/\tau )}{\sum_{j=1}^{N}\exp\left(s\left(t_{i},v_{j}\right)/\tau \right)},\\ L_{\mathrm{align}} & =\frac{1}{2}\left(L_{v\to t}+L_{t\to v}\right). \end{array}$$

Here *N* is the batch size, $\left(v_{i},t_{i}\right)$ is a matched ultrasound–text pair, *j* indexes all text or visual candidates in the batch, *s* is cosine similarity between normalized embeddings, and τ>0 is the temperature. The first term retrieves text given ultrasound; the second retrieves ultrasound given text. This expression illustrates a CLIP-style objective and does not assert that every included alignment model uses identical losses, sampling, or weighting. For separate-encoder (I1) systems, this objective makes independently encoded ultrasound and text representations usable for retrieval and downstream transfer. Its global-pair supervision does not identify the frame or region supporting a clinical phrase, especially when an examination-level report describes findings absent from a sampled frame.

Grounding models, including XBusNet, Grounding DINO-US-SAM, LUS-DET, and VTMedSeg, explicitly connect anatomical terms or text prompts to visual regions [13,20,24,29]. A simplified attention block is

(2) $$\mathrm{Attn}\left(Q,K,V\right)=\mathrm{softmax}\left(\frac{QK^{⊤}}{\sqrt{d}}\right)V.$$

In Equation (2), *Q*, *K*, and *V* are query, key, and value projections of the input tokens, and *d* is the key dimension; which modality supplies each projection depends on the model. This attention operation is not a training objective. In fusion-based (I2) systems, it can condition a class or spatial prediction on text and visual features, while region or mask supervision determines whether the output is locally grounded. Attention weights alone do not establish that a predicted region or generated statement is visually faithful; this requires output-level evaluation against images, annotations, or expert judgments.

Generative systems such as LLAUS, VLS, PlaqueCap, and ECV use the ultrasound representation to condition a language output [11,18,22,32]. A standard autoregressive objective is

(3) $$L_{\mathrm{gen}}=-\sum_{t}\log P\left(y_{t}|x,y_{<t}\right).$$

Here *x* is the visual input, $y_{t}$ is the next reference token, and $y_{<t}$ are preceding text tokens. For ultrasound-conditioned generation (I3), this objective supports flexible answers, captions, and reports, but matching reference wording does not directly penalize a plausible finding that lacks visual support. Text quality metrics therefore need to be supplemented by examination-level factuality checks. Equations (1) and (3) illustrate training objectives, whereas Equation (2) illustrates a fusion operation; none of them alone establishes clinical reliability or permits a cross-architecture performance ranking.

## 4. Representative US-VLMs: Architectures, Learning Paradigms, and Evaluation Evidence

The primary interaction codes organize the 32 proposed systems by the mechanism producing their principal ultrasound endpoint: 10 separate-encoder matching systems, 10 fusion-based prediction systems, and 12 ultrasound-conditioned language generation systems. Learning codes and grounding levels remain separate attributes, rather than alternative architecture families. Table 2 is the operational codebook; Table A1 and Table A2 give study-level assignments and rationales.

### 4.1. Separate-Encoder Matching (I1)

EchoCLIP, EchoPrime, FetalCLIP, and SonoEye learn ultrasound–text matching for representation transfer, retrieval, or diagnosis [14,19,30,31]. FTC-Net uses parallel SigLIP image and text encoders with task-specific LoRA adaptation and similarity-based classification [12]. Sonomate aligns fetal video/text and image/sentence pairs, while FCUFormer aligns view-specific and examination-level representations; their frame/view correspondence is G1 rather than proof of lesion-level localization [15,23]. Prompt-adapted CLIP systems MedLP-HAFB-CLIP, HOPL, and CausCLIP remain I1 when the principal ultrasound result comes from image–text similarity [33,34,35].

### 4.2. Fusion-Based Prediction (I2)

In I2 systems, language enters the prediction path before a class, measurement, box, mask, or anomaly score is produced. XBusNet, CLIP-TNseg, BRC, and VTMedSeg fuse text with visual features for dense segmentation [13,24,36,37]; UltraAD combines image-informed prompts and patch features for anomaly localization [38]. Grounding DINO-US-SAM and LUS-DET localize text-specified anatomy or lesions through a trained box-detection path [20,29]. MMVL and EchoReason use language-guided multimodal representations but obtain their principal diagnosis from classification heads [16,17]. BooF also ends in a trained image–description fusion classifier, although an MLLM supplies an upstream description [39].

### 4.3. Ultrasound-Conditioned Language Generation (I3)

I3 covers the principal trained ultrasound-to-text route, irrespective of whether the language decoder itself is fully fine-tuned. LLAUS, SonoInstruct, MACEE/EEVIT, and VisualGLM pseudo-labeling train visual instruction or attribute-answer generation [11,21,40,41]. AbVLM-Q and the mid-trimester examination system use visually conditioned structured responses for quality assessment [10,42]; HepaCopilot generates a structured reasoning chain that feeds its risk head [43]. PlaqueCap and VLS connect lesion information to generated captions or reports, while ECV and the fuzzy-knowledge model generate ultrasound reports [18,22,32,44]. PMLM includes a visually conditioned diagnosis-text branch alongside unimodal learners, so the study-level taxonomy tables record its hybrid decision path [45]. Generative text quality does not by itself establish that every claim is visually supported.

### 4.4. Temporal Input and Video-Sequence Evidence

Acquisition format and model computation must be distinguished when assessing temporal US-VLMs. Across the 32 studies, four explicitly modeled and evaluated temporal information: EchoPrime, Sonomate, BRC, and HepaCopilot [15,19,37,43]. FetalCLIP evaluated 16-frame clips by concatenating frame embeddings but explicitly did not model fine-grained cardiac dynamics [30]; EchoCLIP trained a two-dimensional image encoder on randomly sampled frames from video–report pairs and identified the absence of a video encoder as a limitation [14]. MMVL and EchoReason describe multi-view ultrasound image sequences, including Doppler inputs, but their reported fusion and ablation analyses do not isolate temporal order from cross-view or cross-modality aggregation [16,17]. FCUFormer aggregates a multi-view image bag, the mid-trimester system reasons over 25 examination thumbnails, and ECV processes cardiac and vascular image windows; these provide examination context without establishing ordered acquisition modeling [10,23,32]. Thus, only 4 of 32 studies (12.5%) met the explicit temporal-evidence rule, one used multi-frame aggregation without fine-grained temporal modeling, one used a video-derived single-frame encoder, two reported sequence inputs without isolating temporal contribution, three used unordered multi-view or examination sets, and the remaining 21 did not provide a temporally resolved model evaluation. These categories describe reported evidence and are not ratings of model quality. Table 3 summarizes temporal-input handling and evidence boundaries.

### 4.5. Task-Stratified Evaluation Evidence

Table 4 groups the 32 studies by principal task and identifies why their numerical outcomes cannot be ranked across studies. For this descriptive cross-tabulation, each included study contributes once, according to the principal task recorded in Table A3 and Table A4 and its primary interaction code assigned in Table A1 and Table A2; secondary tasks do not add further counts. A zero denotes no included study in that task–interaction cell, not an inability of that model type to perform the task. These counts describe research coverage, not capability or performance. Direct numerical comparison requires the same clinical target, evaluation dataset and split, reference standard, unit of analysis, metric definition, and decision threshold when applicable. The included studies were not re-evaluated under such a common protocol. Table A3 and Table A4 retain study-level outcomes together with their reported context and interpretation limits; numerical results there describe within-study evaluations only.

## 5. Datasets, Benchmarks, and Evaluation Protocols

### 5.1. Public and Private Ultrasound Multimodal Datasets

The reviewed studies use a mixture of accessible benchmarks and institution-specific ultrasound cohorts. EchoCLIP and EchoPrime report large video–report sources, while FetalCLIP and SonoInstruct provide fetal and multi-organ image–text training resources [14,19,21,30]. Smaller task datasets support segmentation, risk assessment, visual question answering, and report generation. Dataset scale and independent clinical diversity were analyzed separately. Scale was recorded using unique patients or examinations whenever available; images, frames, videos, reports, masks, boxes, and image–text pairs were treated as nested acquisition or annotation units rather than independent clinical observations. Clinical diversity was based only on directly reported evidence concerning independently sampled institutions or healthcare systems, geographic settings, scanner vendors, operators, acquisition protocols, patient populations, and external test cohorts. Public availability, a large number of acquisition units, or a multicenter label alone was not interpreted as independent clinical diversity. Table 5 summarizes scale and units across organ systems.

Table 6 summarizes reported source breadth and validation independence.

### 5.2. Dataset Bias and Benchmark Limitations

Direct model ranking is hindered by differing case selection, scan units, annotations, splits, and endpoints. Acquisition volume can substantially exceed the number of independent clinical units: EchoCLIP reports 1,032,975 video–report pairs from 99,870 patients, EchoPrime reports 12,124,168 videos from 275,442 studies and 108,913 patients, and FetalCLIP reports 210,035 image–text pairs with its clinical pretraining component drawn from 6493 patients [14,19,30]. These repeated images or videos increase representation coverage but do not create the same number of independent patients. Conversely, FTC-Net is smaller in acquisition volume but draws on 14 institutions and evaluates two independent external cohorts, providing more explicit cross-institution evidence [12].

Source breadth and validation independence describe different properties. EchoPrime evaluates four healthcare systems external to its development system; SonoEye and VLS use two external hospital cohorts; and the fuzzy-knowledge report generator uses organ-specific cohorts from two external hospitals [18,19,31,44]. Grounding DINO-US-SAM holds out three public datasets, which tests cross-dataset transfer but is not equivalent to an independently sampled clinical cohort [29]. AbVLM-Q combines two centers before a random train/validation/test split, so its multicenter source breadth does not constitute external-center validation [42]. Similarly named or reused public datasets were not counted as independent patients, and an external cohort was treated as independent only when it was reported as patient-disjoint, unused in model development or selection, and drawn from a separate institution, healthcare system, or explicitly held-out dataset. Unreported dimensions are marked NR and were not inferred.

### 5.3. Evaluation Metrics Across Tasks

US-VLM evaluation should match the task. Classification commonly uses accuracy, precision, recall, F1-score, and AUC; retrieval uses Recall@K and mean rank; and detection or segmentation uses IoU and Dice, with boundary ambiguity and inter-reader variability limiting interpretation. Report generation and VQA often use BLEU [46], ROUGE [47], METEOR [48], BERTScore [49], CIDEr [50], and exact match [51]. Table 7 links each task family to common metrics and blind spots.

### 5.4. Reporting Standards for US-VLM Studies

US-VLM reports should identify the clinical and acquisition units, organ and task, reference standard, and patient- or examination-level training, validation, and test partitions. They should state how checkpoints, prompts, and thresholds were selected and whether external cohorts remained independent during development. Metrics should include uncertainty intervals and relevant subgroup results; model size, compute and inference latency, code or weight availability, reader evaluation, and representative errors should be reported when applicable. These details make each result interpretable within its own clinical setting.

## 6. Reliability, Explainability, and Safety Evaluation

### 6.1. Failure Modes and Clinical Reliability Risks

US-VLM failures extend beyond generic language hallucination and include errors in anatomical grounding, unsupported findings, quantitative measurements, Doppler interpretation, omission of abnormalities, and diagnostic confidence [8,52]. These failures differ in both mechanism and clinical consequence: generative models may produce unsupported statements, whereas localization or segmentation models more commonly exhibit false-positive, false-negative, or boundary errors. Table 8 summarizes six clinically relevant failure modes identified from the reviewed literature and distinguishes directly reported evidence from indirect signals and currently unreported evaluations. Because most studies do not use a unified case-level safety protocol, the table should be interpreted as an evidence synthesis rather than a ranking of model safety.

### 6.2. Evidence Grounding and Explainability

Beyond identifying failure modes, clinical reliability also requires evidence that model outputs are grounded in the appropriate visual observations. Attention maps and saliency alone may be unstable and weakly correlated with reasoning [53,54]. More auditable approaches include region–text grounding, sentence-level evidence linking, and counterfactual evaluation. Region–text grounding links phrases such as “hypoechoic nodule” to boxes, masks, or pixels. Sentence-level evidence linking connects each generated report sentence to a frame, region, measurement, or cine segment. Counterfactual explanation tests whether changing a prompt, masking a region, or removing a view changes the output consistently. Gaze-based or interaction-based supervision can use eye tracking, cursor trajectories, probe actions, freeze/capture events, zoom/gain adjustments, and measurement operations as weak evidence of expert attention [55,56]. Explanations should state when evidence is incomplete rather than force a rationale [57,58].

### 6.3. Confidence and Uncertainty Estimation

US-VLMs should communicate uncertainty explicitly. Calibration curves and expected calibration error test whether confidence matches empirical accuracy. Selective prediction and abstention allow a model to withhold answers. Confidence intervals can report uncertainty around measurements, segmentation-derived quantities, and risk estimates. Bayesian approximations, ensembles, and conformal prediction can quantify epistemic uncertainty and define prediction sets with coverage guarantees [59,60]. For ultrasound, uncertainty should also reflect acquisition quality, view adequacy, device shift, and anatomical visibility.

### 6.4. Reliability Evaluation Protocol

Automated lexical or task-specific metrics alone do not establish clinical reliability. A practical evaluation protocol should decompose generated answers or reports into atomic clinical statements [61], link visually dependent statements to supporting frames, regions, masks, measurements, or cine segments, assess statement-level confidence and anatomical visibility, and weight errors according to clinical severity [62]. For high-risk applications, these automated assessments should be complemented by expert review. Together with the failure categories in Table 8, this protocol provides a basis for evaluating whether US-VLM outputs are not only accurate, but also evidence-supported and appropriately uncertain.

## 7. Clinical Translation and Deployment

### 7.1. Retrospective Performance and Clinical Evidence

In this review, retrospective task performance means that a model is tested on previously acquired cases, whether the test set comes from the development institution, a public benchmark, or a separate institution. A patient-disjoint external cohort offers stronger evidence of transportability for the specified task than an internal random split, but it remains a retrospective test when the system is not used during care. Expert-created reference labels enable case-level correctness assessment, while expert ratings of model outputs assess judged quality on the sampled cases. A reader study can test how model assistance changes clinicians’ decisions for its participating readers and cases. These forms of evidence answer different questions and should be reported separately.

EchoPrime, FTC-Net, SonoEye, and VLS report external-system or external-hospital results that strengthen retrospective transportability for their specified endpoints [12,18,19,31]. These results do not establish effects on routine decisions or patient outcomes. Such effects require prospective evaluation matched to the intended use, including workflow and safety endpoints when model outputs influence care. Table 9 therefore distinguishes task results, expert input, and remaining clinical evidence gaps.

### 7.2. Intended Use, Risk, and Deployment Readiness

Clinical translation should begin with the intended use of the model. Retrieval and educational applications generally carry lower direct clinical risk than triage, report drafting, risk stratification, or diagnostic assistance, for which an incorrect output may influence downstream management. Validation requirements should therefore be proportional to both intended use and potential harm. Table 9 summarizes the current evidence and remaining deployment gaps across major US-VLM model groups, considering clinical risk, reliability evidence, grounding, and evaluation context. These intended-use groups are descriptive and may overlap; they are not substitutes for the exclusive primary interaction codes in Table 2.

### 7.3. Clinical Validation Pathway

Validation should proceed through clearly identified stages: patient-disjoint internal testing; external testing without using the external cases for model, prompt, or threshold selection; blinded, multi-reader and multi-case assessment of assisted decisions; and prospective evaluation during routine acquisition and reporting [63]. Each stage should state its unit of analysis, reference standard, sampling frame, confidence intervals, and intended-use threshold. External tests should report scanner-vendor, protocol, operator, and site variation [4,64]. Reader studies should compare assisted and unassisted decisions and record inter-reader variability; prospective workflow studies should also record acquisition adequacy, reporting time, management changes, and clinically important errors. Passing one stage does not establish the outcomes tested at a later stage.

### 7.4. Regulatory and Governance Considerations

US-VLMs intended for clinical decision support may fall under software-as-a-medical-device governance. Regulatory logic depends on intended use, risk class, locked versus adaptive behavior, auditability, and post-market monitoring. Governance should define clinician accountability, logging, update policy, incident review, and rollback procedures [65,66]. The included studies do not establish product-level regulatory clearance for the 32 research systems; regulatory status therefore cannot be inferred from these reports. For US-VLMs, governance should also specify when changes in imaging devices, acquisition protocols, prompting strategies, or language-model components require revalidation, particularly when they may affect evidence grounding or generated clinical statements. Human oversight should be explicitly defined for high-risk outputs, including diagnostic conclusions, measurements, and management recommendations.

### 7.5. Practical Deployment Constraints

Deployment requires attention to latency, GPU or edge-device availability, PACS and ultrasound-machine integration, privacy, data locality, hospital workflow, and clinician-in-the-loop interface design. Practical systems may require lightweight alignment, token compression [14], parameter-efficient adaptation, and designs that separate fast perception from slower language generation. Unlike offline image analysis, ultrasound deployment may require models to operate within time-sensitive acquisition workflows, in which image quality and diagnostic views depend on real-time operator interaction. Clinically practical systems should therefore balance multimodal reasoning depth against latency and provide outputs without interrupting image acquisition, measurement, or reporting workflows.

## 8. Open Challenges and Future Roadmap

### 8.1. From Static Inputs to Temporally Grounded Cine- and Exam-Level Reasoning

Only four studies met the explicit temporal-evidence rule, and they addressed different acquisition processes (Table 3). Future work should therefore test clinically relevant temporal information rather than infer it from video input alone.

Sequence models should be compared with matched single-frame and unordered-frame baselines. Frame shuffling, sampling rate, clip length, and temporal coverage can test whether outputs depend on clinically relevant order. Evaluations should match the process under study, such as cardiac motion, CEUS phase transitions, Doppler flow evolution, or sweep progression. Generated findings should identify their supporting frame, segment, view, or measurement; long-sequence systems should also report latency during acquisition.

### 8.2. From Report-Level Alignment to Evidence-Grounded Generation

Because report-level alignment does not identify the image evidence for a finding, future generators should link each clinical statement to a relevant region, frame, measurement, or cine segment. Structured findings could separate perception from narration. Evaluation should test whether the cited evidence actually supports the statement and whether the model expresses uncertainty when evidence is insufficient.

### 8.3. From Private Datasets to Standardized Multicenter Benchmarks

Future benchmarks should identify unique patients and examinations, document center-wise data allocation, and keep external cohorts separate from model, prompt, and threshold selection [67,68]. Standardized metadata should cover scanner, protocol, operator, population, report language, and annotation uncertainty. Shared held-out tests or privacy-preserving multicenter evaluation could support comparisons when raw data cannot be released; results should be reported by site and relevant patient subgroup.

### 8.4. From Accuracy-Only Evaluation to Reliability- and Utility-Oriented Evaluation

Evaluation should predefine clinically important errors, decision thresholds, calibration, and abstention criteria for each intended use. Finding-level correctness and evidence faithfulness should complement task metrics; reader and workflow studies should measure changes in decisions, reporting time, and management rather than infer utility from retrospective AUC alone.

### 8.5. From Offline Models to Governed Clinical Systems

Deployment requires monitoring for changes in scanners, protocols, patient mix, and model components [64,69]. Governance plans should assign responsibility for calibration tracking, incident review, clinician escalation, and revalidation after updates.

## 9. Limitations of This Review

Several limitations should be acknowledged. Considerable heterogeneity across the included studies in clinical tasks, anatomical targets, datasets, model architectures, evaluation protocols, and reported endpoints limited direct quantitative comparison and precluded a meaningful pooled performance estimate. In addition, the methodological appraisal necessarily relied on information reported in the original studies; incomplete reporting of dataset provenance, patient-level partitioning, external validation, or potential data overlap could not always be independently resolved. Finally, because methodological and risk-of-bias judgments were not independently duplicated, some degree of reviewer-level subjectivity cannot be completely excluded. These limitations should be considered when interpreting the synthesized evidence and its generalizability across different ultrasound applications.

## 10. Conclusions

Across 32 studies, US-VLMs support tasks ranging from retrieval and segmentation to diagnostic assistance and report generation. The interaction axis distinguishes separate-encoder matching, fusion-based prediction, and ultrasound-conditioned language generation; learning objectives and grounding granularity add independent information about each system. Reported outcomes cannot support a cross-model ranking because tasks, cohorts, and metrics differ. The risk-of-bias appraisal and dataset analysis also show that large training collections and retrospective task scores provide limited evidence about independent clinical diversity or benefit during care.

Clinical-grade systems will require outputs linked to anatomical and temporal evidence, calibrated uncertainty and appropriate abstention, independent external and prospective validation, and workflow studies that measure effects on clinical decisions and safety. Claims should remain specific to the intended use and to outcomes directly tested.

## Figures and Tables

**Table 1:** Executed three-database search. The search date differs from the database publication cutoff. Bold identifies the total number of records across the three databases.

Database	Search Field	Publication Window	Execution Date	Records
PubMed	Title/Abstract	1 January 2021–30 June 2026	28 September 2026	86
IEEE Xplore	All Metadata	1 January 2021–30 June 2026	28 September 2026	55
Scopus	TITLE-ABS-KEY	1 January 2021–30 June 2026	28 September 2026	263
**Total**	–	–	–	**404**

**Table 2:** Operational three-axis taxonomy for the 32 included studies. I is a single primary interaction code (counts: I1 n=10, I2 n=10, I3 n=12); L codes may be combined; G is the finest documented image–text correspondence, not a performance or faithfulness score. Alternating light-gray rows in this and subsequent tables are used only to aid readability.

Axis/Code	Category	Observable Assignment Criterion	Illustrative Included Systems
I1	Separate-encoder matching	Image/video and text are encoded separately; their embeddings are matched or scored without joint image–text token fusion producing the principal output.	EchoCLIP; Sonomate [14,15]
I2	Fusion-based prediction	Image and text features interact before the principal non-linguistic output (class, value, box, or mask). A language-model backbone with a classification head remains I2.	MMVL; EchoReason [16,17]
I3	Ultrasound-conditioned language generation	An ultrasound-specific trained connector or model conditions generation of the principal textual answer, report, or structured narrative.	LLAUS; VLS [11,18]
L1	Alignment	The included study optimizes ultrasound image/video–text matching or contrastive alignment.	EchoPrime; LUS-DET [19,20]
L2	Supervised output	The included study optimizes a non-linguistic task output against class, box, mask, or measurement labels.	FTC-Net; XBusNet [12,13]
L3	Text generation	The included study trains ultrasound-conditioned text generation, caption/report production, or visual instruction following.	SonoInstruct; PlaqueCap [21,22]
G0	Global pair	The finest documented pair is an image, video, or examination and a class text, report, or answer; local attention alone does not raise this code.	EchoCLIP; FTC-Net [12,14]
G1	Frame/view pair	A frame or named view is explicitly paired with a sentence or descriptor during training or evaluation.	Sonomate; FCUFormer [15,23]
G2	Region/box pair	A lesion, region, or box is explicitly linked to a phrase, caption, coordinate answer, or textual prompt. A frozen mask post-processor does not raise this to G3.	SonoInstruct; VLS [18,21]
G3	Dense output	Text participates in the trained path to a pixel-level anomaly map or segmentation mask, evaluated as such.	XBusNet; VTMedSeg [13,24]

**Table 3:** Temporal-input handling and temporal-evidence assessment in the included studies most relevant to video or sequence reasoning. “Not isolated” means that sequence inputs were described but the contribution of temporal order was not separated from view or modality fusion. Acquisition format alone was not treated as evidence of temporal modeling. Alternating light-gray rows in this and subsequent tables are used only to aid readability.

Study	Actual Model Input	Order Status	Temporal Mechanism or Evaluation	Interpretation Boundary
EchoPrime [19]	16-frame cine clips and multiple videos per study	Retained	mVIT video encoder; frame-shuffle sensitivity; single-frame, single-video, and multi-video comparisons	Models cine dynamics and examination aggregation; report statements are not localized to supporting frames.
Sonomate [15]	Freehand fetal videos sampled at 5 frames/s with transcribed audio	Retained	Video–text alignment; timestamped frame–sentence alignment with context correction; sequence VQA	Temporal evidence concerns scan progression and audio–video alignment; visual features are extracted frame-wise.
BRC [37]	Echocardiography sequences spanning the cardiac cycle	Retained	Three-dimensional convolutional encoding; text-guided temporal consistency; cardiac-size trend error	Supports sequence segmentation consistency rather than open-ended video report generation.
HepaCopilot [43]	16 frames from each of three CEUS phases (48 total)	Retained	Phase-aware attention, cross-phase attention, single-frame ablation, and temporal-aggregator comparisons	The test set contained 18 cases; external frame-count and sampling sensitivity were not established.
FetalCLIP [30]	16-frame fetal-heart clips	Aggregated	Frame embeddings concatenated for a linear CHD probe; averaging and concatenation compared	The study states that fine-grained cardiac dynamics were not explicitly modeled.
EchoCLIP [14]	Randomly sampled frames from A4C videos	Discarded	ConvNeXt image encoder aligned with report text; no video encoder	Video–report provenance does not make the trained visual pathway temporal.
MMVL [16]	Multi-view B-mode, color-Doppler, and spectral-Doppler sequences	Not isolated	Domain encoders, flattened visual tokens, and cross-modality gating; no reported frame-order test	Reported gains cannot be attributed specifically to temporal information.
EchoReason [17]	Multi-view cardiac and vascular ultrasound sequences	Not isolated	Expert encoders and spatial-token fusion; no reported temporal ablation	Parameter and diagnostic results combine view, modality, and sequence information.

**Table 4:** Task-stratified evaluation evidence for the 32 included studies. I1/I2/I3 entries are numbers of studies in the three operational interaction categories defined in Table 2; each row total equals the corresponding study count, and column totals are 10/10/12. Study-level outcomes and citations appear in Table A3 and Table A4. Alternating light-gray rows in this and subsequent tables are used only to aid readability.

Principal Task Family	Studies	I1/I2/I3	Reported Endpoint Types	Main Comparison Boundary
Representation/retrieval	4	4/0/0	Retrieval rank; downstream transfer or measurement endpoints	Different target tasks, pairing units, and test sets.
Diagnosis/risk prediction	9	4/3/2	AUC, accuracy, F1, or sensitivity	Different organs, disease definitions, cohorts, and decision thresholds.
Scan quality/standard planes	4	2/0/2	Plane accuracy, examination F1, or quality scores	Image-, plane-, and examination-level units are not interchangeable.
Detection/segmentation/anomaly localization	7	0/7/0	Detection, Dice/IoU, or anomaly-map endpoints	Different organs, annotation standards, datasets, and spatial units.
Report/caption generation	4	0/0/4	Text metrics, expert judgments, or task-specific diagnosis endpoints	Different report targets, reference texts, and factuality assessments.
Visual QA/instruction following	4	0/0/4	Answer accuracy, attribute scores, or within-study gains	Different questions, answer spaces, and benchmark construction.

**Table 5:** Dataset scale and acquisition units across organ systems. Unique patients, participants, examinations, cases, or records are separated from nested images, videos, reports, instructions, and pairs. NR denotes not reported; counts are not additive across studies. Alternating light-gray rows in this and subsequent tables are used only to aid readability.

Organ	Dataset/Cohort	Access	Independent Clinical Units	Nested Acquisition/Annotation Units	Primary Use
Breast	BLU (BrEaST)	Public	NR	252 analyzed images; lesion masks	XBusNet segmentation [13]
Breast	BUSI/BUSZS	Public/constructed	NR	Images and lesion labels; source counts kept distinct	Few-shot and anomaly studies [38]
Breast	BUS-BRA	Public	NR	1875 images (1268 benign, 607 malignant)	BooF training and evaluation [39]
Breast	BUS-UCLM	Public	NR	683 images (419 normal, 174 benign, 90 malignant)	UltraAD adaptation [38]
Breast	GDPH&SYSUCC benchmark + separate institutional set	Public + institutional	1202 benchmark patients; institutional patient count NR	2405 benchmark images; 204 separate institutional images	HOPL few-shot BI-RADS classification [34]
Breast	Hangzhou breast nodule cohort	Institutional	3100 cases (2217 benign, 883 malignant)	Image–report units; number NR	PMLM diagnosis [45]
Thyroid	FTC-Net multicenter cohort	Institutional	2421 patients	6477 images	Diagnosis across 14 institutions [12]
Thyroid	PKTN	Public	NR	1005 images	CLIP-TNseg training with DDTI and web data [36]
Fetal	FetalCLIP pretraining data	Study-specific	6493 patients in the clinical component	210,035 image–text pairs, including textbook pairs	Contrastive pretraining [30]
Fetal	Planes DB (FetalCLIP use)	Public	1792 patients	Image count not specified for this evaluation	FetalCLIP plane classification and probing [30]
Fetal	HC18	Public	663 + 161 patients, as reported	814 test images	FetalCLIP gestational-age and segmentation tests [30]
Fetal	Fetal Planes DB (other uses of the same resource)	Public	1762 cases in the mid-trimester pretraining subset; Sonomate test patients NR	12,400 mid-trimester pretraining images; 5271 Sonomate external-test images	Mid-trimester CNN pretraining; Sonomate external test [10,15]
Fetal	SonoInstruct/SonoBench	Constructed benchmark	NR	110k+ images; 260k+ instructions	Instruction tuning and benchmark evaluation [21]
Fetal	FCUFormer cohort	Institutional	1669 patients in the original cohort; final patient count NR	5495 final JPEG images with reports	Multi-view image–report learning [23]
Fetal	FOCUS	Public	NR	300 images	FCUFormer external classification test [23]
Fetal	Sonomate examinations	Study-specific	525 examinations/scan pairs	525 video–audio pairs (456/14/55 split)	Scan-level video–language grounding [15]
Fetal	MedLP Level II cohort	Institutional	1261 pregnant women	6137 images	Standard-plane classification [33]
Cardiac (portable)	P-Echo	Institutional	NR	5146 images (350 few-shot; 4796 test)	CausCLIP quality assessment [35]
Cardiac	C-Echo	Institutional	NR	16,572 images	CausCLIP source-domain fine-tuning [35]
Cardiac	EchoCLIP source corpus	Institutional	99,870 patients; 224,685 studies	1,032,975 video–report pairs	Video–text pretraining [14]
Cardiac	EchoPrime source corpus	Institutional development	108,913 patients; 275,442 studies	12,124,168 videos with reports	Multi-view whole-study alignment [19]
Cardiac	EchoNet-Dynamic	Public	NR in this synthesis	10,030 echocardiography sequences	EchoCLIP external test; BRC segmentation [14,37]
Cardiac	CAMUS	Public	500 patients	Echocardiography sequences; count NR	BRC segmentation [37]
Cardiac	HMC-QU	Public	140 A4C patients + 100 A2C patients; overlap NR	Echocardiography sequences; count NR	BRC segmentation [37]
Cardiac	Cardiac UDA	Public	Approximately 100 patients	410 sequences	BRC segmentation [37]
Cardiac	BRC private cohorts	Institutional	178 adult + 100 pediatric patients	Echocardiography sequences; count NR	BRC segmentation [37]
Cardiac	ECED	Constructed knowledge base	Not a patient cohort	Text, tables, diagrams, and image-centric instructions	Echo MLLM expertise tuning [40]
Cardiac + vascular	MMVL cohort	Institutional	1922 patients/examinations	Multi-view sequences; count NR	Cardiovascular diagnosis [16]
Cardiac + vascular	EchoReason cohort	Institutional	2277 patients	Multi-view sequences; count NR	Measurement-guided diagnosis [17]
Cardiovascular	ECV paired records	Study-specific	11,276 paired patient records	335,151 images	Report and measurement tasks [32]
Coronary (IVUS)	MICCAI 2011 IVUS challenge	Public	10 subjects	2175 images	PlaqueCap captioning [22]
Abdominal	AbVLM-Q cohort	Two-center institutional	7766 scans	Images used as scan units	Standard-plane quality assessment [42]
Liver	LUSP/LUDD	Study-specific/public	NR	LUSP: 13,720 images, 44,669 boxes; LUDD: 1367 images	LUS-DET pretraining and detection [20]
Liver (CEUS)	TCIA B-mode-and-CEUS-Liver	Public	120 patients (84/18/18 split)	Multiphase CEUS videos/frames; count NR	HepaCopilot risk stratification [43]
Ophthalmic	VLS three-hospital cohort	Institutional	9670 patients	64,098 images; 21,355 reports	Grounded report generation [18]
Ophthalmic	SonoEye cohort	Institutional	70,452 development patients; 941 internal-test; 511 + 1666 external	215,356 development image–text pairs	Risk stratification [31]
Endoscopic	VisualGLM EUS cohort	Institutional	519 cases	7021 images	Multi-attribute pseudo-label generation [41]
Multi-organ	18 US segmentation sets	Public	Patient counts vary/NR	15 adaptation sets; 3 held-out sets (2808 images)	Grounding DINO-US-SAM segmentation [29]
Multi-organ	BUSI/DDTI/TN3K/Prostate micro-US	Public	Patient counts vary/NR	Images and masks	VTMedSeg segmentation [24]
Multi-organ	BUSM/USQA/MUSC	Public/released	NR	10k caption pairs; 660 QA pairs; 249 captioning pairs	LLAUS tuning and evaluation [11]
Multi-organ reports	Public US report corpus	Public	7390 cases	Two representative images and a report per case	Report generation training [44]
Multi-organ reports	Sun Yat-sen hospital cohort	Institutional	15,000 cases (5000 per organ)	Representative images and reports	Report generation training [44]
Multi-organ reports	External validation cohorts	Institutional	1421 cases (499 thyroid; 498 liver; 424 breast)	Images and reports	Cross-hospital report evaluation [44]

**Table 6:** Reported evidence of independent clinical diversity and external validation in representative multi-source studies. The table records observable source separation rather than assigning a diversity score. “External” is used only for a cohort reported as separate from model development or selection. Alternating light-gray rows in this and subsequent tables are used only to aid readability.

Study/Cohort	Development-Source Breadth	Independent Test Evidence	Reported Diversity Dimensions	Interpretation Boundary
EchoPrime [19]	One development healthcare system; 108,913 patients	Four external healthcare systems; international test sources	System, country, view, and video-count variation	Strong cross-system evidence; development volume still contains repeated videos and studies per patient.
FTC-Net [12]	14 institutions; 1462 development patients	Two external cohorts: 578 and 381 patients	Institution and device subgroup analyses	Multicenter development and two patient-level external validations are separately reported.
SonoEye [31]	Site A development cohort; no external data used in training	Sites B and C: 511 and 1666 patients	Site, age subgroup, and multi-view patient aggregation	Independent retrospective external sites; prospective workflow validation remains absent.
VLS [18]	SAHZU development, validation, and internal test cohorts	FAHWM: 269 patients; FAHZC: 70 patients	Hospital, age, sex, disease mix, images, and reports	Two external hospitals are explicit, but both external cohorts are much smaller than development data.
Fuzzy-knowledge report model [44]	Public corpus plus 15,000 cases from one development hospital	Two external hospitals: 499 thyroid, 498 liver, and 424 breast cases	Hospital, organ, disease, and age-group coverage	Cross-hospital and cross-organ testing; external cohorts remain organ-specific.
Mid-trimester VLM [10]	273 internal examinations plus public pretraining data	29 examinations from a second institution	Institution and examination-level protocol	External-institution evidence is present but based on a small examination sample.
Grounding DINO-US-SAM [29]	15 public adaptation datasets	Three held-out public datasets (2808 images)	Dataset, organ, and annotation-source heterogeneity	Demonstrates cross-dataset transfer, not independently recruited clinical validation.
AbVLM-Q [42]	7766 scans from two hospitals and several scanner vendors	No external-center test; pooled random 70/15/15 split	Hospital and vendor breadth	Multicenter source breadth is reported, but center independence is not tested.
VisualGLM EUS [41]	519 cases and 7021 images from five centers	No center-held-out test reported; pooled 60/20/20 split	Center and missing-attribute patterns	Five-center collection increases source breadth without establishing external-center generalization.
EchoCLIP [14]	One institutional source corpus; 99,870 patients	EchoNet-Dynamic used for task-specific external evaluation	Institution and public-benchmark separation	External evidence applies to specified downstream endpoints, not to every capability of the source model.

**Table 7:** Evaluation metrics and their interpretation across task types in the 32 reviewed studies. Examples are within-study observations. Most studies rely at least partly on institution-specific, often non-public cohorts; differences in tasks, datasets, splits, and metrics preclude direct performance comparisons. Alternating light-gray rows in this and subsequent tables are used only to aid readability.

Task	Representative Studies	Common Metrics	Within-Study Example	What Metrics Capture	What They Miss
VQA/visual dialogue	SonoInstruct; LLAUS; EEVIT [11,21,40]	Accuracy; task score	SonoInstruct: 30.3% relative gain	Answer correctness	Visual evidence and clinical harm
Report generation	VLS; PlaqueCap; ECV [18,22,32]	BLEU; ROUGE; expert rating	VLS: internal BLEU-4 66.37	Text similarity	Unsupported statements
Retrieval/representation	EchoCLIP; EchoPrime; FetalCLIP [14,19,30]	Recall@K; AUC; MAE	EchoPrime: video–text Recall@10 98%	Embedding/task performance	Case-level clinical utility
Classification/diagnosis	FTC-Net; SonoEye; FCUFormer [12,23,31]	AUC; accuracy; F1	FTC-Net: external AUC 0.836/0.841	Discrimination	Calibration and shift
Risk/decision support	HepaCopilot; FTC-Net (versus TI-RADS risk stratification) [12,43]	AUC; sensitivity; specificity	HepaCopilot: AUROC 0.94 (n = 18)	Case-level classification	Prospective decision impact
Segmentation/grounding	XBusNet; VLS; BRC [13,18,37]	Dice; IoU; detection	XBusNet: Dice 0.8766 on BLU	Mask agreement	Report-level factuality
Anomaly detection	UltraAD [38]	Image/pixel AUROC	Three breast US datasets evaluated	Anomaly ranking	Lesion-level false negatives
Quality/standard planes	AbVLM-Q; CausCLIP; mid-trimester VLM [10,35,42]	Accuracy; F1; quality score	AbVLM-Q: Excellent-grade (8–10) scoring accuracy 85.11%; plane classification 98.96%	View/exam scoring	Live operator behavior

**Table 8:** Failure modes and evidence from the 32 reviewed studies. Direct, proxy and unquantified signals are distinguished in the supporting-evidence column. Alternating light-gray rows in this and subsequent tables are used only to aid readability.

Failure Mode	Definition	Supporting Evidence	Clinical Impact	Mitigation and Gap
Wrong anatomy/spatial alignment	Wrong organ, region, view or lesion assigned to text.	Direct evidence: AbVLM-Q reports landmark/region detection errors; XBusNet reports mask-level errors [13,42]	Wrong finding location or view.	Check phrase-to-region, view and exam correspondence.
Unsupported generated finding	Generated claim lacks evidence in the US input.	Direct evidence: SonoInstruct analyzes clinical hallucination in generated reports; BooF identifies spurious MLLM descriptions; PlaqueCap and VLS add lesion grounding [18,21,22,39]	Unnecessary work-up or treatment.	Require expert-audited statement-level factuality.
False size/extent	Boundary or quantitative extent differs from reference.	Proxy evidence: XBusNet and BRC report mask or segmentation errors; neither establishes a general report-measurement error rate [13,37]	Wrong lesion threshold or ventricular boundary decision.	Measure numerical error against readers, not only masks.
Unsupported Doppler statement	Vascular flow claim lacks Doppler support.	Not quantified as a dedicated case-level error in the included reports; vascular ultrasound report tasks are evaluated in ECV, and CEUS tasks in HepaCopilot [32,43]	Incorrect vascular interpretation.	Evaluate Doppler-specific factuality and abstention.
Missed abnormality	True lesion or feature omitted or missed.	Direct proxy: XBusNet reports false-negative pixels; VLS reports per-disease sensitivities as low as 0% for uveal melanoma, and HOPL reports 33.33% sensitivity for BI-RADS 5 [13,18,34]	False reassurance or delayed diagnosis.	Report lesion and patient-level false-negative rates.
Uncalibrated certainty	Confidence does not track diagnostic correctness.	Partial direct evidence: HepaCopilot reports ECE and Brier metrics with temperature scaling; FTC-Net and SonoEye report calibration curves; MedLP-HAFB-CLIP flags low-confidence predictions for manual review [12,31,33,43]	Over-reliance on erroneous answer.	Assess calibration, selective prediction and model-specific thresholds.

**Table 9:** Clinical risk, reliability and deployment-readiness checklist for model groups represented in the 32-paper corpus. Risk labels are narrative categories, not regulatory classifications. Retrospective task results, expert input, and prospective effects on care are distinct evidence types. Alternating light-gray rows in this and subsequent tables are used only to aid readability.

Model Group	Intended Use	Clinical Risk	Reliability/Safety Evidence	Explainability/Grounding	Evidence Context	Deployment Gap
LLM-adapted US assistants	Image-grounded QA	Medium–High	SonoBench and echo VQA tasks evaluated [21,40]	Instruction/image pairing	Task-specific expert input	Prospective clinical utility
Report generators	Draft US reports	High	VLS and PlaqueCap report task metrics [18,22]	Mask/lesion prompts in some studies	Study-specific expert review	Statement-level factuality
Segmentation/grounding	Lesion or organ masks	Medium	Dice/IoU across task cohorts [13,29]	Masks and text-guided regions	Annotation-based	Cross-device clinician utility
Contrastive US encoders	Retrieval/representation	Low–Medium	EchoCLIP, FetalCLIP task evaluations [14,30]	Embedding similarity	Retrospective validation	Clinical calibration/shift
Few-shot anomaly models	Abnormality screening	Medium–High	UltraAD breast benchmarks [38]	Anomaly maps	Not a prospective reader trial	External lesion-level safety
Quality-control models	Plane/exam scoring	Medium	AbVLM-Q and mid-trimester study include quality results [10,42]	Plane and structure signals	Six sonographers in mid-trimester study [10]	Live scanning validation
Echo-specific VLMs	Echo interpretation	Medium–High	EchoCLIP, EchoPrime, EchoReason evaluated [14,17,19]	Video/view/measurement evidence	Retrospective multisystem results	Workflow impact trial
US risk-stratification models	Thyroid/eye/liver risk	High	FTC-Net, SonoEye, HepaCopilot metrics [12,31,43]	Image–text and risk outputs	Cohort-specific reference labels	Prospective threshold safety

## Data Availability

No new participant-level data were generated. Study-level extraction, taxonomy assignments, and risk-of-bias judgments for all 32 included articles are reported in the text and Table A1, Table A2, Table A3 and Table A4.

## Abbreviations

The following abbreviations are used in this manuscript:

VLM	Vision–Language Model
US-VLM	Ultrasound Vision–Language Model
LLM	Large Language Model
MLLM	Multimodal Large Language Model
CLIP	Contrastive Language–Image Pretraining
VQA	Visual Question Answering
RG	Report Generation
IC	Image Classification
ITR	Image–Text Retrieval
OD	Object Detection
IS	Image Segmentation
MAT	Multimodal Alignment Technique
PEFT	Parameter-efficient Fine-tuning
LoRA	Low-Rank Adaptation

## Appendix A. Complete Search Strategies

The database-specific queries below reproduce the search strings executed on 28 September 2026 with a 30 June 2026 publication cutoff. Search fields and publication-period restrictions are stated separately for clarity. Listings A1–A3 reproduce the PubMed, IEEE Xplore, and Scopus queries, respectively.

### Appendix A.1. PubMed

**Search execution date:** 28 September 2026
**Search field:** Title/Abstract
**Publication period:** 1 January 2021–30 June 2026
**Records retrieved:** n=86
**Complete search string:**

**Listing A1.** PubMed search string as executed on 28 September 2026.
(ultrasound[Title/Abstract] OR ultrasonograph*[Title/Abstract] OR sonograph*[Title/Abstract] OR echograph*[Title/Abstract] OR echocardiograph*[Title/Abstract] OR POCUS[Title/Abstract] OR ``point-of-care ultrasound’’[Title/Abstract] OR ``point of care ultrasound’’[Title/Abstract] OR ``endoscopic ultrasound’’[Title/Abstract] OR endosonograph*[Title/Abstract] OR EUS[Title/Abstract] OR ``intravascular ultrasound’’[Title/Abstract] OR IVUS[Title/Abstract] OR ``contrast-enhanced ultrasound’’[Title/Abstract] OR CEUS[Title/Abstract] OR ``fetal ultrasound’’[Title/Abstract] OR ``foetal ultrasound’’[Title/Abstract] OR ``transvaginal ultrasound’’[Title/Abstract] OR ``transcranial ultrasound’’[Title/Abstract] OR ``US imaging’’[Title/Abstract] OR ``US image’’[Title/Abstract] OR ``US video’’[Title/Abstract] OR ``US-guided’’[Title/Abstract]) AND ((``vision-language’’[Title/Abstract] OR ``vision language’’[Title/Abstract] OR ``visual-language’’[Title/Abstract] OR ``visual language’’[Title/Abstract] OR VLM[Title/Abstract] OR VLMs[Title/Abstract] OR ``multimodal large language model’’[Title/Abstract] OR ``multimodal large language models’’[Title/Abstract] OR ``multi-modal large language model’’[Title/Abstract] OR ``multi-modal large language models’’[Title/Abstract] OR MLLM[Title/Abstract] OR MLLMs[Title/Abstract] OR ``large multimodal model’’[Title/Abstract] OR ``large multimodal models’’[Title/Abstract] OR ``large multi-modal model’’[Title/Abstract] OR ``large multi-modal models’’[Title/Abstract] OR ``multimodal language model’’[Title/Abstract] OR ``multimodal language models’’[Title/Abstract] OR ``multi-modal language model’’[Title/Abstract] OR ``multi-modal language models’’[Title/Abstract] OR ``multimodal foundation model’’[Title/Abstract] OR ``multimodal foundation models’’[Title/Abstract] OR ``multi-modal foundation model’’[Title/Abstract] OR ``multi-modal foundation models’’[Title/Abstract] OR ``image-text model’’[Title/Abstract] OR ``image text model’’[Title/Abstract] OR ``image-language model’’[Title/Abstract] OR ``image language model’’[Title/Abstract] OR ``vision-text model’’[Title/Abstract] OR ``visual-text model’’[Title/Abstract]) OR (CLIP[Title/Abstract] AND (``contrastive language-image’’[Title/Abstract] OR ``contrastive language image’’[Title/Abstract] OR ``CLIP model’’[Title/Abstract] OR ``CLIP encoder’’[Title/Abstract] OR ``CLIP pretraining’’[Title/Abstract] OR ``vision-language’’[Title/Abstract] OR ``image-text’’[Title/Abstract])) OR (MedCLIP[Title/Abstract] OR BioMedCLIP[Title/Abstract] OR ``BLIP-2’’[Title/Abstract] OR LLaVA[Title/Abstract] OR ``GPT-4V’’[Title/Abstract] OR GPT4V[Title/Abstract] OR ``GPT-4o’’[Title/Abstract] OR ``Qwen-VL’’[Title/Abstract] OR InternVL[Title/Abstract] OR MiniGPT-4[Title/Abstract] OR ``Med-Flamingo’’[Title/Abstract] OR ``DeepSeek-VL’’[Title/Abstract])) AND (``2021/01/01’’[Date - Publication] : ``2026/06/30’’[Date - Publication])

### Appendix A.2. IEEE Xplore

**Search execution date:** 28 September 2026
**Search field:** All Metadata
**Publication period:** 1 January 2021–30 June 2026 (record-level cutoff check applied)
**Records retrieved:** n=55
**Complete search string:**

**Listing A2.** IEEE Xplore search string as executed on 28 September 2026.
(``All Metadata’’:ultrasound OR ``All Metadata’’:ultrasonograph* OR ``All Metadata’’:sonograph* OR ``All Metadata’’:echograph* OR ``All Metadata’’:echocardiograph* OR ``All Metadata’’:POCUS OR ``All Metadata’’:``point-of-care ultrasound’’ OR ``All Metadata’’:``point of care ultrasound’’ OR ``All Metadata’’:``endoscopic ultrasound’’ OR ``All Metadata’’:endosonograph* OR ``All Metadata’’:EUS OR ``All Metadata’’:``intravascular ultrasound’’ OR ``All Metadata’’:IVUS OR ``All Metadata’’:``contrast-enhanced ultrasound’’ OR ``All Metadata’’:CEUS OR ``All Metadata’’:``fetal ultrasound’’ OR ``All Metadata’’:``foetal ultrasound’’ OR ``All Metadata’’:``transvaginal ultrasound’’ OR ``All Metadata’’:``transcranial ultrasound’’ OR ``All Metadata’’:``US imaging’’ OR ``All Metadata’’:``US image’’ OR ``All Metadata’’:``US video’’ OR ``All Metadata’’:``US-guided’’) AND ((``All Metadata’’:``vision-language’’ OR ``All Metadata’’:``vision language’’ OR ``All Metadata’’:``visual-language’’ OR ``All Metadata’’:``visual language’’ OR ``All Metadata’’:VLM OR ``All Metadata’’:VLMs OR ``All Metadata’’:``multimodal large language model’’ OR ``All Metadata’’:``multimodal large language models’’ OR ``All Metadata’’:``multi-modal large language model’’ OR ``All Metadata’’:``multi-modal large language models’’ OR ``All Metadata’’:MLLM OR ``All Metadata’’:MLLMs OR ``All Metadata’’:``large multimodal model’’ OR ``All Metadata’’:``large multimodal models’’ OR ``All Metadata’’:``large multi-modal model’’ OR ``All Metadata’’:``large multi-modal models’’ OR ``All Metadata’’:``multimodal language model’’ OR ``All Metadata’’:``multimodal language models’’ OR ``All Metadata’’:``multi-modal language model’’ OR ``All Metadata’’:``multi-modal language models’’ OR ``All Metadata’’:``multimodal foundation model’’ OR ``All Metadata’’:``multimodal foundation models’’ OR ``All Metadata’’:``multi-modal foundation model’’ OR ``All Metadata’’:``multi-modal foundation models’’ OR ``All Metadata’’:``image-text model’’ OR ``All Metadata’’:``image text model’’ OR ``All Metadata’’:``image-language model’’ OR ``All Metadata’’:``image language model’’ OR ``All Metadata’’:``vision-text model’’ OR ``All Metadata’’:``visual-text model’’) OR (``All Metadata’’:CLIP AND (``All Metadata’’:``contrastive language-image’’ OR ``All Metadata’’:``contrastive language image’’ OR ``All Metadata’’:``CLIP model’’ OR ``All Metadata’’:``CLIP encoder’’ OR ``All Metadata’’:``CLIP pretraining’’ OR ``All Metadata’’:``vision-language’’ OR ``All Metadata’’:``image-text’’)) OR (``All Metadata’’:MedCLIP OR ``All Metadata’’:BioMedCLIP OR ``All Metadata’’:``BLIP-2’’ OR ``All Metadata’’:LLaVA OR ``All Metadata’’:``GPT-4V’’ OR ``All Metadata’’:GPT4V OR ``All Metadata’’:``GPT-4o’’ OR ``All Metadata’’:``Qwen-VL’’ OR ``All Metadata’’:InternVL OR ``All Metadata’’:MiniGPT-4 OR ``All Metadata’’:``Med-Flamingo’’ OR ``All Metadata’’:``DeepSeek-VL’’))

### Appendix A.3. Scopus

**Search execution date:** 28 September 2026
**Search field:** TITLE-ABS-KEY
**Publication period:** 1 January 2021–30 June 2026 (record-level cutoff check applied)
**Records retrieved:** n=263
**Complete search string:**

**Listing A3.** Scopus search string as executed on 28 September 2026.
TITLE-ABS-KEY(ultrasound OR ultrasonograph* OR sonograph* OR echograph* OR echocardiograph* OR POCUS OR ``point-of-care ultrasound’’ OR ``point of care ultrasound’’ OR ``endoscopic ultrasound’’ OR endosonograph* OR EUS OR ``intravascular ultrasound’’ OR IVUS OR ``contrast-enhanced ultrasound’’ OR CEUS OR ``fetal ultrasound’’ OR ``foetal ultrasound’’ OR ``transvaginal ultrasound’’ OR ``transcranial ultrasound’’ OR ``US imaging’’ OR ``US image’’ OR ``US video’’ OR ``US-guided’’) AND (TITLE-ABS-KEY(``vision-language’’ OR ``vision language’’ OR ``visual-language’’ OR ``visual language’’ OR VLM OR VLMs OR ``multimodal large language model’’ OR ``multimodal large language models’’ OR ``multi-modal large language model’’ OR ``multi-modal large language models’’ OR MLLM OR MLLMs OR ``large multimodal model’’ OR ``large multimodal models’’ OR ``large multi-modal model’’ OR ``large multi-modal models’’ OR ``multimodal language model’’ OR ``multimodal language models’’ OR ``multi-modal language model’’ OR ``multi-modal language models’’ OR ``multimodal foundation model’’ OR ``multimodal foundation models’’ OR ``multi-modal foundation model’’ OR ``multi-modal foundation models’’ OR ``image-text model’’ OR ``image text model’’ OR ``image-language model’’ OR ``image language model’’ OR ``vision-text model’’ OR ``visual-text model’’) OR TITLE-ABS-KEY(CLIP AND (``contrastive language-image’’ OR ``contrastive language image’’ OR ``CLIP model’’ OR ``CLIP encoder’’ OR ``CLIP pretraining’’ OR ``vision-language’’ OR ``image-text’’)) OR TITLE-ABS-KEY(MedCLIP OR BioMedCLIP OR ``BLIP-2’’ OR LLaVA OR ``GPT-4V’’ OR GPT4V OR ``GPT-4o’’ OR ``Qwen-VL’’ OR InternVL OR MiniGPT-4 OR ``Med-Flamingo’’ OR ``DeepSeek-VL’’)) AND PUBYEAR > 2020 AND PUBYEAR < 2027

## Appendix B. Study-Level Taxonomy Assignments and Risk-of-Bias Appraisal

Table A1 and Table A2 apply the operational taxonomy rules in Section 2.6 and the separate risk-of-bias rules in Section 2.4 to all 32 studies in the same order. Interaction is the primary image–text computation for the principal ultrasound endpoint; L codes may coexist; G is the finest documented image–text relation and does not certify clinical faithfulness. Descriptions name the mechanism that determined each assignment. In particular, a frozen SAM or other downstream mask generator does not convert a trained text-to-box pathway into G3, and a language-model backbone feeding a class head is coded I2. The seven-character RoB profile lists D1–D7 in order; each character is L, H, or U as defined in Section 2.4. Taxonomy codes and RoB judgments answer different questions.

**Table A1:** Study-level taxonomy assignments and risk-of-bias judgments, studies 1–16. I/L/G codes are defined in Table 2; the RoB profile lists D1–D7 in the order defined in Section 2.4. Overall L/H/U judgments are scoped to the reported endpoint; the final column gives the basis for each overall judgment.

Study	I	L	G	Assignment Rationale	RoB D1–D7	Basis for Overall Judgment
MedLP-HAFB-CLIP [33]	I1	L2	G0	Learnable text prompts scored against whole-image CLIP features for plane classification.	LLLULUU	U: Whether development and test images overlap and how uncertainty was assessed were not reported.
SonoInstruct/SonoBench [21]	I3	L3	G2	Ultrasound visual instruction tuning includes SonoGrounding box-coordinate answers.	UUULLLU	U: Patient overlap among the public sources was not reported.
FetalCLIP [30]	I1	L1	G0	Paired fetal image–text representation pretraining with contrastive matching.	UULULUL	U: Reference provenance varies by task.
SonoEye [31]	I1	L1+L2	G0	Image–text contrastive pretraining followed by patient-label fine-tuning.	ULLLLLL	U: Diagnostic cohort is enriched.
HepaCopilot [43]	I3	L2+L3	G0	CEUS features condition autoregressive reasoning; a head uses its hidden state for risk prediction.	LLLLLUL	U: Only 18 held-out patients.
Mid-trimester VLM [10]	I3	L3	G1	Images plus CNN-generated plane descriptions condition fine-tuned examination-level responses.	ULLULHL	H: Fixed order of assisted reader phases.
FTC-Net [12]	I1	L2	G0	Parallel SigLIP encoders compare images with FTA/FTC class text; LoRA is tuned with focal loss.	LLLLLLL	L: Histology; external patient cohorts.
VLS [18]	I3	L3	G2	VLM generates reports and lesion boxes; the boxes prompt an unfine-tuned SAM mask stage.	ULUULLL	U: Whether the within-site split was patient-level was not reported.
XBusNet [13]	I2	L2	G3	Clinical text modulates global/local visual branches that predict lesion masks.	LLLLLLL	L: One scan/patient; expert masks; CV.
PlaqueCap [22]	I3	L2+L3	G2	Lesion segmentation supplies a spatial prompt to Q-Former/frozen LLM captioning.	ULUUUUL	U: Ten subjects; a disjoint test set was not reported.
EchoPrime [19]	I1	L1+L2	G0	Video–text contrastive training and a supervised view classifier support study-level interpretation.	LULLLLL	U: Report-derived reference varies by task.
Grounding DINO-US-SAM [29]	I2	L2	G2	LoRA-tuned text-guided detector predicts boxes; downstream SAM2 remains frozen.	UUUULUL	U: Patient grouping within each dataset was not reported.
AbVLM-Q [42]	I3	L3	G2	Qwen2-VL outputs structure coordinates and quality judgments under spatial prompts.	ULUULUL	U: Scans were split at random after pooling; patient grouping was not reported.
BRC [37]	I2	L1+L2	G3	Multilevel image–text pretraining feeds text-guided attention and a mask decoder.	UUUULUL	U: Patient-level allocation across splits was not reported.
EchoCLIP [14]	I1	L1	G0	Echocardiogram video and report encoders use contrastive matching.	LULLLLL	U: Report-derived reference targets.
Sonomate [15]	I1	L1	G1	Coarse video–text and finer image–sentence alignment support fetal scan understanding.	UULULUL	U: Annotation independence varies by task.

**Table A2:** Study -level taxonomy assignments and risk-of-bias judgments, studies 17–32 (continued from Table A1).

Study	I	L	G	Assignment Rationale	RoB D1–D7	Basis for Overall Judgment
HOPL [34]	I1	L2	G0	Ordinal/class-specific text prompts are scored with CLIP image embeddings.	ULUULLL	U: Whether the image split grouped patients was not reported.
FCUFormer [23]	I1	L1+L2	G1	Separate text encoder aligns each fetal cardiac view and pooled view bag with descriptions.	UULULUL	U: How abnormality labels were adjudicated was not reported.
VisualGLM pseudo-labeling [41]	I3	L3	G0	Prompt-tuned VisualGLM produces ultrasound tumor attribute answers/pseudo-labels.	UUUULUL	U: Whether images from the same case were separated across splits was not reported.
UltraAD [38]	I2	L2	G3	Image-informed text prompts fuse with patch tokens for anomaly maps and classification.	UUUUUUL	U: Patient grouping and case-selection criteria were not reported.
VTMedSeg [24]	I2	L2	G3	Synthetic geometry text and image features enter concept-aware fusion for mask prediction.	LUULLLL	U: Patient grouping of the public splits was not reported.
PMLM [45]	I3	L2+L3	G0	Multimodal learner generates diagnosis text; final decision also uses two unimodal learners.	LLUULLL	U: Whether cases were split patient-wise was not reported.
MACEE/EEVIT [40]	I3	L3	G0	Echo expertise is incorporated by visual instruction tuning for VQA.	UUULLUL	U: Patient identities within the reused benchmarks were not reported.
LUS-DET [20]	I2	L1+L2	G2	Contrastive pretraining precedes text-injected feature fusion and box detection.	ULUULUL	U: Image-level split; patient overlap between splits was not reported.
EchoReason [17]	I2	L2	G0	Prompt/measurement-enhanced shared representation yields regression and disease-class logits, not generated prose.	LLLULUL	U: Potential overlap between development sources was not reported.
ECV [32]	I3	L2+L3	G0	Modality-specific visual experts feed measurement and ultrasound report decoders.	UULULUL	U: Report factuality reference not adjudicated.
Fuzzy-knowledge MLLM [44]	I3	L3	G0	LLaVA-OV-based generator integrates fuzzy knowledge for ultrasound reports.	UUUULUL	U: Whether the internal split was patient-wise was not reported.
LLAUS [11]	I3	L3	G0	Ultrasound visual instruction tuning with Zoom-In produces image-conditioned answers.	UHULLUL	H: Generated test answers lack expert check.
CLIP-TNseg [36]	I2	L2	G3	FiLM fuses CLIP text/image features before the pixel-wise thyroid mask head.	ULUULUL	U: Patient-level partitioning was not reported.
MMVL [16]	I2	L2	G0	Joint visual/prompt tokens yield a fused [CLS] state and disease-class logits.	LLLLLUL	U: Uncertainty intervals for outcomes were not reported.
CausCLIP [35]	I1	L1+L2	G0	CLIP scoring is adapted with causal visual features and multigranular image–text constraints.	ULUULLL	U: Image split lacks patient grouping.
BooF [39]	I2	L2	G0	MLLM descriptions feed a trained attention-gated image–text diagnostic classifier.	LLUULUL	U: Whether cross-validation folds were patient-disjoint was not reported.

## Appendix C. Study-Level Outcomes and Evaluation Context

Table A3 and Table A4 present task-specific outcomes for the 32 included studies. Reported study data or scale may describe the training corpus or overall cohort and must not be read as the denominator of a particular test result. An endpoint is described without a single numerical value when no common summary result is suitable for this cross-task overview. Within-study baseline comparisons apply only to their stated evaluation setting. Most studies rely at least partly on institution-specific, often non-public cohorts, and their tasks, evaluation protocols, and metrics differ; none of the rows supplies a cross-study performance ranking.

**Table A3:** Study -level reported outcomes, studies 1–16.

Study/Year	Principal Task	Reported Study Data/Scale	Within-Study Endpoint or Finding	Interpretation Boundary
MedLP-HAFB-CLIP [33] 2026	Fetal standard-plane recognition	1261 pregnant women	100% accuracy in reported observational experiment	Observational subset; task-specific
SonoInstruct/SonoBench [21] 2026	US visual QA/reasoning	110k+ images; 260k+ instructions	30.3% relative improvement versus base model	Benchmark-specific comparison
FetalCLIP [30] 2026	Fetal representation and transfer	210,035 paired fetal US images	Task-specific fetal transfer comparisons reported	No single common task metric
SonoEye [31] 2026	Eye disease risk stratification	215,356 pairs; 70,452 patients	98.3% screening sensitivity	Eye-specific risk categories
HepaCopilot [43] 2026	HCC risk stratification	TCIA liver CEUS cohort	AUROC 0.94; 95% CI 0.82–1.00	Wide confidence interval in paper
SonoNet + fine-tuned VLM [10] 2026	Mid-trimester exam quality	22,544 planes; 273 internal + 29 external examinations	External F1 up to 0.922	Exam-level quality task
FTC-Net [12] 2026	FTC versus FTA diagnosis	2421 patients; 14 institutions	External AUCs 0.836 and 0.841	Follicular-neoplasm population
VLS [18] 2026	Ophthalmic US reporting	64,098 images; 21,355 reports	Internal diagnostic accuracy 90.59%	External accuracy differs
XBusNet [13] 2025	Breast lesion segmentation	BLU breast US dataset	Mean Dice 0.8766 on BLU	Single-cohort cross-validation
PlaqueCap [22] 2025	Plaque captioning/localization	Curated IVUS plaque cohort	Task-specific baseline comparisons reported	IVUS plaque-specific evidence
EchoPrime [19] 2026	Whole-echo interpretation	Over 12 M video-report pairs	Video–text Recall@10 98%; multiple task-specific benchmarks	Multiple endpoints; no common metric
Grounding DINO-US-SAM [29] 2025	Multi-organ segmentation	18 public US datasets; 3 held out	Results reported across seen and held-out datasets	Heterogeneous dataset endpoints
AbVLM-Q [42] 2025	Standard-plane quality assessment	7766 abdominal US scans	Plane label accuracy 98.96%	Quality scoring, not diagnosis
BRC [37] 2025	Label-efficient echo segmentation	Echocardiography segmentation data	Task-specific segmentation results reported	Task-specific masks
EchoCLIP [14] 2024	Echo retrieval/LVEF	1,032,975 echo videos + reports	LVEF MAE 8.4% internal; 7.1% external (EchoNet-Dynamic)	Retrospective benchmark endpoints
Sonomate [15] 2026	Fetal scan understanding	Freehand fetal US examinations	Expert and benchmark evaluations reported	Fetal examination setting

**Table A4:** Study-level reported outcomes, studies 17–32 (continued from Table A3).

Study/Year	Principal Task	Reported Study Data/Scale	Within-Study Endpoint or Finding	Interpretation Boundary
HOPL [34] 2026	Few-shot breast classification	Breast US BI-RADS cohorts	Few-shot classification results reported	BI-RADS task
FCUFormer [23] 2026	Fetal cardiac abnormality diagnosis	5495 final images; 1669 patients in original cohort	Average diagnostic accuracy 87.37%	Fetal-cardiac task; final patient count NR
VisualGLM pseudo-labeling [41] 2026	Attribute diagnosis/pseudo-labels	Endoscopic US tumor cohort	Attribute accuracy about 0.85 with missing input	Pseudo-labeling task
UltraAD [38] 2025	Anomaly localization/classification	Three breast US datasets	Task-specific baseline comparisons reported	Few-shot breast setting
VTMedSeg [24] 2026	Multi-organ segmentation	US segmentation datasets	Task-specific segmentation results reported	Synthetic text descriptions
PMLM [45] 2026	Breast nodule diagnosis	Breast nodule images + text	Task-specific classification results reported	Task-specific breast cohort
MACEE/EEVIT [40] 2025	Echo VQA	ECED: 100+ cardiac conditions	Echo VQA task-specific outcomes reported	Knowledge-enhancement evaluation
LUS-DET [20] 2026	Open-vocabulary liver detection	44,669 region-caption pairs	Zero-shot detection reported	Anatomy-to-lesion transfer
EchoReason [17] 2026	Cardiovascular diagnosis	Cardiac + vascular US cohort	Measurement and diagnosis endpoints reported	Multistage task-specific design
ECV MoE framework [32] 2026	Cardiac/vascular report generation	Over 10k paired US records	Report and measurement endpoints reported	Multi-domain US task
Fuzzy-knowledge MLLM [44] 2025	Multi-organ report generation	Breast, liver, thyroid US reports	Report-generation metric results reported	Generated-report factuality not pooled
LLAUS [11] 2025	Ultrasound VQA	US captions and QA corpus	Reported VQA results	Instruction-data dependent
CLIP-TNseg [36] 2025	Thyroid nodule segmentation	Thyroid US segmentation data	Task-specific mask-overlap results reported	Thyroid-only evaluation
MMVL [16] 2025	Cardiovascular diagnosis	Cardiac + vascular US	10.9% reported accuracy gain	Conference task-specific study
CausCLIP [35] 2024	Quality/view assessment	Portable echocardiography	Absolute gains 4.1%, 9.5%, 8.5% across tasks	Portable echo domain shift
BooF [39] 2026	Breast malignancy diagnosis	Breast US datasets	Task-specific diagnostic results reported	Expert-feedback pipeline
